# *Schistosoma mansoni* infection among preschool age children attending Erer Health Center, Ethiopia and the response rate to praziquantel

**DOI:** 10.1186/s13104-019-4246-8

**Published:** 2019-04-05

**Authors:** Makida Kemal, Gemechu Tadesse, Adem Esmael, Solomon Mequanente Abay, Tadesse Kebede

**Affiliations:** 1 School of Medicine, Goba Referral Hospital, Madda Walabu University, Bale Robe, Ethiopia; 2 Ethiopian Public Health Institute, Addis Ababa, Ethiopia; 3 Department of Pharmacology and Clinical Pharmacy, School of Pharmacy, College of Health Sciences, Addis Ababa University, Addis Ababa, Ethiopia; 4 Department of Microbiology Immunology and Parasitology, School of Medicine, College of Health Sciences, Addis Ababa University, Addis Ababa, Ethiopia

**Keywords:** *S. mansoni*, Praziquantel, Pre-school aged children, Ethiopia

## Abstract

**Objective:**

Preschool age children (PSAC) are excluded from community based praziquantel treatment programs mainly due to paucity of evidence on the magnitude of schistosomiasis, efficacy and safety of this treatment in PSAC. The aim of this study is to assess *Schistosoma mansoni* infection rate and evaluate response to praziquantel in PSAC. A facility based longitudinal study was employed from April to June 2016 at Erer Health Center, Eastern Ethiopia. Stool sample was examined for schistosomiasis in 236 PSAC and repeated after 4 weeks post-treatment in positive individuals. Treatment outcomes were recorded and interpreted.

**Results:**

Out of the 236 study participants, 59 (25%) were infected with *S. mansoni*. Praziquantel treatment (40 mg/kg) resulted in 96.4% cure rate and 99.4% egg reduction rate. Children of 3–5 year old were significantly affected with *S. mansoni* infection. Nausea and fatigue were common mild adverse events within 4 h of treatment however moderate and severe adverse events and allergic reactions were not observed. In conclusion, praziquantel at 40 mg/kg, the dose utilized in standard care for school age children, is tolerable and efficacious in the treatment of *S. mansoni* infection in PSAC, which calls for the healthcare system to provide appropriate service for this population.

**Electronic supplementary material:**

The online version of this article (10.1186/s13104-019-4246-8) contains supplementary material, which is available to authorized users.

## Introduction

Schistosomiasis is one of the most common parasitic diseases in many parts of Ethiopia, usually at an altitude between 1200 and 2000 m above sea level [1]. The anthelmintic drug praziquantel is the cornerstone for morbidity control with millions of people treated every year [2, 3]. It has been a drug of choice for schistosomiasis control for more than 40 years [4]. However, preschool age children (PSAC) are currently excluded from preventive chemotherapy control campaigns. The reasons for this exclusion include: PSAC to be considered at low risk of infection, insufficiency of evidence in safety and efficacy of praziquantel for this age group [5] and lack of a pediatric formulation [6]. Recently, there is growing of evidence of infection in infants and young children in different parts of sub-Saharan African countries [7]. For instance a community based cross-sectional study in Northwest Ethiopia showed that 11.2% of PSAC are infected with *S. mansonia* [8]. The reported disease burden in this specific age group raise a concern of how the existing standard treatment of school aged children is extended to PSAC in high risk areas with the optimized dose of praziquantel.

Many studies showed that treatment with praziquantel is tolerable and efficacious in school age children (SAC) for the treatment of schistosomiasis [9]. Due to the limited evidence on the safety for children under 5 years of age at the moment, preventive chemotherapy for schistosomiasis is not indicated for this age group in many countries [10]. Although PSAC may also be at risk of schistosomiasis similar to SAC and can be infected very early in childhood, to authors’ knowledge the efficacy and tolerability for praziquantel in the treatment of *Schistosoma mansoni* infection in young children in Ethiopia was not assessed. So, this study was aimed to assess *S.* *mansoni* infection in PSAC and the response rate for praziquantel treatment (40 mg/kg).

## Main text

### Methods

#### Study design

A facility based longitudinal study was employed. A facility based screening and follow-up were arranged to ensure that the PSAC, minor study participants, are under proper standard care.

#### Study site and study population

The study was conducted at Erer Woreda Health Center, Shinile Zone of Somali Region, Ethiopia from April to June 2016 having catchment population of 77,628 at Erer district according to national census in 2007 [11]. The average elevation of this district is 824 meters above sea level [12]. In this district, more than 89.6% of housing units get drinking water from well, spring, river and lake. River is one of the main source of drinking water with easy access to children main [11].

All PSAC who visited Erer Health Center for different medical reasons during the study period were included. A total of 236 children were screened for *S. mansoni* eggs. Those participants who are positive for the test were treated with praziquantel 40 mg/kg and followed for a month to assess the treatment outcome.

#### Inclusion and exclusion criteria

*Inclusion criteria* PSAC less than 6 years of age, whose parents gave written informed consent for the study.

*Exclusion criteria* PSAC who were treated with praziquantel within a month time of study recruitment period.

#### Data collection techniques and clinical assessments

All demographic and clinical information were recorded on a standard record form. Interviews were conducted by nurses at the Health Center. Information on health condition of children and adverse events after treatment were included in the interview. Mothers/guardians of children were interviewed at 4 and 24 h post treatment to assess tolerability of praziquantel.

#### Stool sample collection and laboratory procedures

Stool samples were collected initially once per day for two consecutive days and on day 28 and day 29 for re-assessment to evaluate drug response. Parasitological diagnosis of *S. mansoni* was performed using double Kato-Katz thick smears preparation at the Health Center Laboratory. The number of *S. mansoni* eggs were counted and recorded.

#### Dosing and treatment procedure

Children with positive stool examination for *S. mansoni* who were treated with the standard single dose praziquantel prepared by trained nurses according to weight-based guideline (40 mg/kg) were considered for follow-up. At each supervised drug administration by the health care providers, children were observed for 30 min.

#### Follow-up procedures

Children who were enrolled in the study and treated with single dose of praziquantel were appointed for clinical assessments. Evaluations of adverse events were performed with parent/guardian report using standard list of praziquantel related adverse events at 4 and 24 h post-treatment follow-up. Stool examination was performed to determine egg reduction rate (ERR) and cure rate (CR) of praziquantel using the following formulas:

$${\text{Cure rate}} = \left( {1 - \frac{{{\text{Number of }}S. mansoni\,{\text{negative participants}}}}{{{\text{Number of }}S. mansoni \,{\text{positive participants}}}}} \right) \times 100$$

$${\text{ERR}} = \left( {1 - \frac{\text{Geometric mean of egg per gram after treatment}}{\text{Geometric mean of egg pergram before treatment}}} \right) \times 100$$

#### Statistical analysis

Data were recorded on standardized record forms and double entered to EPI data 3.1 and exported to SPSS software version 20 for analysis. Descriptive statistics were calculated for variables and bivariate analysis was conducted to check statistically significant association between variables. Geometric mean egg counts were calculated for participants who are positive for *S. mansoni*. It was categorized into infection intensities: light infections, 1–99 epg, moderate infections: 100–399 epg; and heavy infections: > 400 epg according to WHO guidelines [10].

### Results

#### Socio-demographic characteristics of the participant

The mean age of 236 PSAC children included in the study was 3.38 (Standard deviation ± 1.05) years, and majority of the study participants were at age 4 year [n = 72, 30.5%], 121 were girls (51.3%) and majority were from rural kebeles [n = 197, 83.5%]. Most of mothers/care givers of the study participants were 18–27 years old [n = 103, 43.6%], house-wife [n = 178, 75.4%] and unable to write and read in educational status [n = 103, 43.6%] (Tables 1 and 2).

**Table 1:** Socio-demographic characteristics of preschool age children

Category	*S. mansoni* infection		OR (95% CI)		P-value
	No. positive (%)	Total^a^ (%)	Crude	Adjusted	
Age (years)					
2	6 (9.7)	62 (26.3)	1	1	
3	18 (29.0)	62 (26.3)	3.82 (1.39, 10.43)	5.05 (1.72, 14.80)	0.003
4	22 (30.6)	72 (30.5)	4.11 (1.54, 10.94)	4.65 (1.67, 12.49)	0.003
5	13 (32.5)	40 (16.9)	4.49 (1.54, 13.11)	5.70 (1.86, 17.55)	0.002
Total	59 (25)	236			
Sex					
Boy	32 (27.8)	115 (48.7)	1.34 (0.74, 2.42)	1.60 (0.84, 3.03)	0.149
Girl	27 (22.3)	121 (51.3)	1	1	
Residence					
Urban	7 (17.9)	39 (16.5)	1	1	
Rural	52 (26.4)	197 (83.5)	1.64 (0.68, 3.94)	2.26 (0.78, 6.63)	0.133

^a^Total number of participants examined (%)

**Table 2:** Socio-demographic characteristics of parent/guardian of preschool age children

Category	*S. mansoni* infection		OR (95% CI)		P-value
	No. Positive (%)	Total^a^ (%)	Crude	Adjusted	
Age group					
18–27	27 (26.2)	103 (43.6)	1	1	
28–37	19 (25.3)	75 (31.8)	1.23 (0.58, 2.62)	1.16 (0.52, 2.67)	0.725
38 and above	13 (22.4)	58 (24.6)	1.17 (0.52, 2.63)	1.17 (0.49, 2.77)	0.728
Educational status					
Illiterate	24 (23.3)	103 (43.6)	2.17 (0.25, 18.15)	0.51 (0.19, 1.31)	0.160
Read and write	23 (24.7)	93 (39.4)	2.30 (0.27, 19.69)	0.52 (0.19, 1.34)	0.175
Formal education	12 (30.0)	40 (16.9)	1	1	
Ethnicity					
Somali	32 (27.6)	116 (49.6)	1		
Oromo	23 (23.2)	99 (42.3)	0.71 (0.31–1.60)	0.77 (0.39–1.50)	0.446
Amhara	4 (21.1)	19 (8.1)	0.77 (0.33, 1.75)	0.59 (0.73, 2.02)	0.401
Marital status					
Married	56 (25.9)	216 (91.5)	1.43 (0.35, 5.90)	1.27 (0.27, 5.91)	0.765
Others	3 (15.0)	20 (8.5)	1	1	
Occupation					
Housewife	47 (26.4)	178 (75.4)	0.73 (0.35, 1.49)	1.034 (0.23, 4.63)	0.999
Others^b^	12 (20.7)	58 (24.6)	1	1	
Frequency of using river/spring or pond water for bathing a child					
Not at all	16 (23.2)	69 (29.2)	1	1	
Sometimes	32 (23.7)	135 (57.2)	1.03 (0.52, 2.04)	1.22 (0.55, 2.72)	0.627
Often	11 (34.4)	32 (13.6)	1.74 (0.69, 4.35)	2.15 (0.69, 6.65)	0.183
Frequency of taking children to river/pond during fetching water					
Not at all	27 (25.5)	106 (44.9)	1	1	
Sometimes	26 (21.7)	120 (50.8)	0.81 (0.44, 1.49)	0.74 (0.37, 1.47)	0.384
Often	6 (60.0)	10 (4.2)	4.39 (1.15, 16.74**)**	3.86 (1.86, 17.28)	0.044

^a^Total number of participants examined (%)

^b^Others (merchant, employed and others)

#### *Schistosoma mansoni* infection and associated factors

Bivariate analysis was made for each explanatory variable of *S. mansoni* infection. Age, sex, address and frequency of taking children to river or pond during fetching water fulfill minimum condition for association. According to bivariate and multivariate analysis; age of children was significantly associated with *S. mansoni* infection in PSAC. Children at 5 year old age category were the most significantly associated with *S. mansoni* infection with [AOR = 5.70, 95% CI (1.86, 17.50), p = 0.002]. The more often children taken to river during fetching water were statistically associated with higher *S. mansoni* infection rate [AOR = 3.86, 95% CI (1.86, 17.28), p = 0.044] (Tables 1 and 2).

#### *Schistosoma mansoni* infection rate

Among PSAC screened for *S. mansoni,* 59 (25%) were positive (Table 1). Of the 59 infected patients, 24 (40.7%) had light and 35 (59.3%) moderate *S. mansoni* infection. The overall mean egg count before treatment was 142.25 epg. The moderate intensity of *S. mansoni* infection in relation to sex was slightly higher among males 18 (30.5%) than in females 17 (28.8%) (Table 3). Only two cases were positive for *S. mansoni* 4 weeks post treatment. The pre-treatment geometric mean egg count for *S. mansoni* infection in PSAC was 204.92 epg and reduced to 1.18 epg 4 weeks after treatment.

**Table 3:** Intensity of *Schistosoma mansoni* among preschool age children

Variables	Light (1–99 epg)	Moderate (100–399 epg)	Total
Age (years)			
2	4 (6.8%)	2 (3.4%)	6 (10.2%)
3	8 (13.56%)	10 (16.9%)	18 (30.46%)
4	7 (11.86%)	15 (25.4%)	22 (37.26%)
5	5 (8.5%)	8 (13.56%)	13 (22.06%)
Sex			
Male	14 (23.72%)	18 (30.5%)	32 (54.22%)
Female	10 (16.94%)	17 (28.81%)	27 (45.75%)

#### Cure rate and egg reduction rate

Cure rate after treatment was calculated as the percentages of individuals who were tested as *S. mansoni* egg free after treatment. The CR and the ERR were 96.4% and 99.42% respectively.

#### Tolerability of praziquantel

Most of the adverse events were observed within the first 4 hs after treatment. In this period, majority of the treated study participants experienced mild adverse events that include nausea [84.7%], fatigue [84.7%] and abdominal pain [72.9%] (Additional file 1: Table S1).

### Discussion

In the present study, prevalence of *S. mansoni* egg positivity among PSAC attending Erer Health center was 25%. A cure rate of 96.4% was achieved by treating the PSAC that were found positive for schistosomiasis with the standard care intervention, praziquantel 40 mg/kg. The egg reduction rate was 99.42%.

The institution based prevalence of *S. mansoni* among PSAC was 25%. This finding is similar to reports from cross-sectional survey conducted in Azaguie district of Côte d’Ivoire where the prevalence of *S. mansoni* among PSAC was 21.6% (n = 160) [13]. The prevalence of *S. mansoni* egg positivity in Hamad Alla village, Sudan was 44.3% (n = 106) [14] and in Uganda studied in 7 lakeshore villages on 5 months to 7 year-old children was 37.7% (n = 979) [15]. From a targeted epidemiological survey in Uganda, egg positivity rate was found to be 44.3% (n = 131) at Lake Albert and 16% (n = 232) at Lake Victroria areas [16]. A cross sectional household based study conducted in Wondo Genet, Southern Ethiopia reveals that 37.2% of *S. mansoni* infection in under-five children [17]. The difference in rate of positivity among the reports might be due to difference in sampling population, study design and geographical settings.

The present study showed that children at the age of five were more likely infected with *S. mansoni* compared to lower age groups. A similar study conducted in Ugandan PSAC showed that children aged between 3 and 6 years had higher risk of infection than their younger counterparts [16]. This might be due to high frequency of water body contact with the aforementioned age groups. The current study shows that children who were often taken to river during fetching water were three times more likely infected with *S. mansoni* compared to those not taken at all.

In the current study praziquantel achieved CR of 96.4% against *S. mansoni* infections 4 weeks after treatment among PSAC. Cure rate of this study was higher than that of study conducted in Uganda 77.6% [15], study in districts of Côte d’Ivoire 88.6% [5] and Sudan 90.5% [14]. We found that praziquantel ERR after 4 weeks of treatment in PSAC was 99.4%. Whereas, a study conducted in Uganda with a follow up of 3–4 weeks showed ERR of 99.1% [15] and Cote d Ivoire with a follow up period of 3 weeks was 96.7% [5]. The differences in ERR and CR might be due to difference in the time to evaluate treatment efficacy, the presence of a large number of immature worms, intensity of transmission and intensities of pretreatment egg loads.

In conclusion, the study revealed that a quarter of screened PSAC are positive for *S. mansoni* infection. PSAC had low or moderate *S. mansoni* infection intensity, which responds to crushed praziquantel at a dose of 40 mg/kg with CR of 96.4%. Though the study supports use of praziquantel in PSAC as recommended by WHO for preventive chemotherapy program in SAC, confirmatory large scale clinical studies are required to identify pharmacokinetic and pharmacodynamic determinants to improve patient outcomes.

## Limitation of the study

The main limitation is that samples come from children that attend the health facility, and therefore extrapolating the percentage infected to the general population as a whole is unjustified.

## Additional file

**Additional file 1: Table S1.** Reported adverse events within 4 and 24 h post-treatment of praziquantel.

## Abbreviations

AOR: adjusted odds ration
CR: cure rate
epg: eggs per gram
ERR: egg reduction rate
PSAC: pre-school age children
SAC: school age children
WHO: World Health Organization
