# Supplementary material for: Schistosoma mansoni infection among preschool age children attending Erer Health Center, Ethiopia and the response rate to praziquantel
Source: BMC Res Notes. 2019 Apr 5;12:211. doi: 10.1186/s13104-019-4246-8 (PMC6451229; doi:10.1186/s13104-019-4246-8)
Supplement: Supplementary file 1 — Additional file 1: Table S1. Reported adverse events within 4 and 24 h post-treatment of praziquantel. [file 13104_2019_4246_MOESM1_ESM.docx]

**Additional file 1: Table S1. Reported adverse events within 4 and 24 hs post-treatment of praziquantel**

| **Mild adverse events** | **Within 4 hs, n (%)** | **Within 24 hs, n (%)** |
| --- | --- | --- |
| Nausea | 50 (84.7) | 15 (25.4) |
| Vomiting | 13 (22.03) | - |
| Headache | 36 (61.01) | 13 (22.03) |
| Fatigue | 50 (84.7) | 3 (57.6) |
| Abdominal pain | 43 (72.9) | 8 (13.5) |
| Stomachache | 10 (16.9) | 2 (3.9) |
| Sweating | 4 (6.8) | - |
| Mild fever | 1. (6.8) | - |

*Excluding the report during the first 4 hrs.
